# Catecholaminergic and cholinergic neuromodulation in autism spectrum disorder: A comparison to attention-deficit hyperactivity disorder

**DOI:** 10.3389/fnins.2022.1078586

**Published:** 2023-01-06

**Authors:** Damian Koevoet, P. K. H. Deschamps, J. L. Kenemans

**Affiliations:** ^1^Experimental Psychology, Helmholtz Institute, Utrecht University, Utrecht, Netherlands; ^2^Department of Psychiatry, University Medical Center Utrecht, Utrecht, Netherlands

**Keywords:** dopamine, norepinephrine, acetylcholine, cerebellum, Purkinje cells, genetics, salience network, biomarkers

## Abstract

Autism spectrum disorder (ASD) is a heterogeneous neurodevelopmental disorder characterized by social impairments and restricted, repetitive behaviors. Treatment of ASD is notoriously difficult and might benefit from identification of underlying mechanisms that overlap with those disturbed in other developmental disorders, for which treatment options are more obvious. One example of the latter is attention-deficit hyperactivity disorder (ADHD), given the efficacy of especially stimulants in treatment of ADHD. Deficiencies in catecholaminergic systems [dopamine (DA), norepinephrine (NE)] in ADHD are obvious targets for stimulant treatment. Recent findings suggest that dysfunction in catecholaminergic systems may also be a factor in at least a subgroup of ASD. In this review we scrutinize the evidence for catecholaminergic mechanisms underlying ASD symptoms, and also include in this analysis a third classic ascending arousing system, the acetylcholinergic (ACh) network. We complement this with a comprehensive review of DA-, NE-, and ACh-targeted interventions in ASD, and an exploratory search for potential treatment-response predictors (biomarkers) in ASD, genetically or otherwise. Based on this review and analysis we propose that (1) stimulant treatment may be a viable option for an ASD subcategory, possibly defined by genetic subtyping; (2) cerebellar dysfunction is pronounced for a relatively small ADHD subgroup but much more common in ASD and in both cases may point toward NE- or ACh-directed intervention; (3) deficiency of the cortical salience network is sizable in subgroups of both disorders, and biomarkers such as eye blink rate and pupillometric data may predict the efficacy of targeting this underlying deficiency *via* DA, NE, or ACh in both ASD and ADHD.

## Introduction

Autism spectrum disorder (ASD) is a neurodevelopmental disorder characterized by behavioral (including social) deficits (American Psychiatric Association, 2013). In the DSM-5 the two foremost criteria for ASD, often referred to as the core deficits, are social deficits and restricted and repetitive behaviors. In addition to these core deficits, individuals with ASD suffer from a wide range of impairments. Examples of such impairments are deficits in learning (Klinger et al., 2007; Schreibman et al., 2015), executive function (Craig et al., 2016), language (Tager-Flusberg, 2006; Edgar et al., 2015), aggression (Kanne and Mazurek, 2011), hyperactivity (Lecavalier, 2006), sleeping (Malow et al., 2006; Deserno et al., 2019), and sensory processing (Marco et al., 2011). Many view ASD as a very heterogeneous disorder, partly due to the great variability in the presentation as well as severity of symptoms (Masi et al., 2017). While high-functioning groups can – at least in part – effectively participate in society, low-functioning ASD groups show higher severity of symptoms (e.g., no verbal communication) and cannot function independently (Lord et al., 2004; Whitby and Mancil, 2009; Baghdadli et al., 2012; Masi et al., 2017). Due to the great heterogeneity of ASD in addition to the complex nature of social communication and interaction, it is hard to identify effective biological/pharmacological treatments for ASD. To date, there are no effective medications that treat the core deficits of ASD and many treatments tend to focus on other symptoms such as irritability (Coury, 2010).

As to the underlying neurobiological correlates, ASD has been linked to impaired function or structure of prefrontal cortex (PFC; Carper and Courchesne, 2005; Stoner et al., 2014; Zhu et al., 2015), amygdala (Baron-Cohen et al., 2000), cerebellum (Becker and Stoodley, 2013; Hampson and Blatt, 2015), insula (Uddin and Menon, 2009; Kosaka et al., 2010), basal ganglia (Nayate et al., 2005), as well as numerous other brain regions (Stoner et al., 2014). Others have linked the dysfunction of broader systems or networks to ASD. Examples are the broken mirror neuron hypothesis and the dysfunction of the salience network (SaN; Ramachandran and Oberman, 2006; Uddin and Menon, 2009). However, neither of these hypotheses have been able to consistently account for the wide range of underlying neurobiological correlates of ASD [i.e., see Southgate and Hamilton (2008) for a criticism on the broken mirror neuron hypothesis; Müller, 2007]. Thus, the focus on only one or a group of neural structures has not led to a unifying hypothesis of ASD, as no consistent pathology has emerged for the disorder – no genetic or neurobiological factors are very consistently present in ASD, nor are they specific when compared to other psychiatric disorders (Müller, 2007; Amaral et al., 2008; de la Torre-Ubieta et al., 2016). Müller (2007) suggests that ASD should be viewed as a distributed disorder in which most — if not all — brain networks are affected. Therefore, examining connectivity between and within brain regions and neural networks could yield a more comprehensive picture of the neurobiological deficits underlying ASD.

Connectivity within well-defined networks in relation to ASD has been an area of great interest (e.g., Mizuno et al., 2006; Kleinhans et al., 2008; Monk et al., 2009; Anderson et al., 2011; Wass, 2011; Nair et al., 2013; Uddin et al., 2013; Kana et al., 2014; Nomi and Uddin, 2015; Duan et al., 2017). Sridharan et al. (2008) suggest that the SaN is responsible for switching between the activation of the default-mode network (DMN) and executive control networks (ECN; Seeley et al., 2007; Menon and Uddin, 2010). Whereas the DMN is often regarded as the resting-state network that activates when no task is being performed, the ECN activates during the performance of tasks (Seeley et al., 2007; Sridharan et al., 2008; Toro et al., 2008; Raichle, 2015). Some authors suggest that the anterior insula, an important structure in the SaN, is aberrantly connected in ASD (Uddin and Menon, 2009; Ebisch et al., 2011; Guo et al., 2019). Similar ideas have been proposed for the DMN and ECN (Murdaugh et al., 2012; Moseley et al., 2015; Farrant and Uddin, 2016). Nevertheless, results from over- and under-connectivity investigations are — likely in part due to methodological problems and the heterogeneity of ASD cohorts — inconsistent and reproducibility has been limited (Müller et al., 2011).

Understanding neural connectivity is an important first step in identifying how the brain effectuates important functions. However, a connection diagram of the brain should indeed be seen as a first step, and not as the final answer to comprehending how nervous systems realize function. One potential complication, as evidenced in animal studies, is that connectivity is dynamic and can change over time (Brezina, 2010; Marder, 2012; Bargmann and Marder, 2013). Such dynamic changes over time occur across several years in interaction with the environment (Lawrence et al., 2019), but also within a matter of (milli)seconds (Marder, 2012). Almost all current human ASD studies have assessed connectivity as being static within a single functional magnetic resonance imaging (fMRI) scanning session, while such connections are likely dynamic (Brezina, 2010; Marder, 2012; Falahpour et al., 2016). One study did consider these dynamic changes when studying individuals with ASD. Using fMRI, Falahpour et al. (2016) compared conventional ‘static’ scanning procedures with ‘dynamic’ scanning procedures that take into account physiological change during the scanning period (Allen et al., 2014). Using the static procedure, previous findings were replicated: the ASD group showed aberrant connectivity in the DMN. In contrast, the dynamic scanning procedure showed that the ASD group not differ from control subjects in peak connectivity but did show greater intraindividual variability of functional connectivity during the scanning period. Thus, connections in ASD are not ‘broken’ but show higher levels of intraindividual variability across time (Falahpour et al., 2016; London, 2018). Variability of connectivity, as opposed to under- or over-connectivity, may contribute to the pathology of ASD. It is therefore important to consider what drives the variability of connectivity. One obvious perspective is the role of the classic ascending modulating neurotransmitter systems, or neuromodulatory systems.

Neuromodulation is the altering of activity within a neural network by electrical, mechanical, or chemical means (Krames et al., 2009).^1^ Nervous systems have a wide range of such neuromodulatory systems (Brezina, 2010; Marder, 2012). This review focuses on the three classic neuromodulatory systems: NE from the locus coeruleus (LC), DA from the ventral tegmental area (VTA) and the substantia nigra pars compacta (SNc), and acetylcholine (ACh) from the nucleus basalis of Meynert (NBM; Andeìn et al., 1966; Foote and Morrison, 1987; McCormick, 1989, 1992; Mesulam, 2000; Aston-Jones and Cohen, 2005a; Stahl, 2008). These ascending projections have widespread potent and sustained–or more transient (Aston-Jones and Cohen, 2005b; e.g., Aston-Jones and Cohen, 2005a; Schutte et al., 2020)–modulatory effects on neurotransmission in the forebrain (Andeìn et al., 1966; Foote and Morrison, 1987; McCormick, 1989, 1992; Robbins, 2000; Stahl, 2008; Marder, 2012). In addition, focusing on these three systems in relation to ASD also provides a window on potential underlying mechanisms that overlap with those disturbed in other developmental disorders, for which treatment options are more obvious. The prime example is attention-deficit hyperactivity disorder (ADHD), given the efficacy of especially stimulants in treatment of ADHD (Wilens, 2008; Huss et al., 2017).

Indeed, neuromodulatory neurotransmitters have been of particular interest in relation to attention-deficit hyperactivity disorder (ADHD; Robbins, 2000; Robbins and Arnsten, 2009; del Campo et al., 2011). ADHD is a neurodevelopmental disorder characterized by hyperactivity, impulsivity and inattention (American Psychiatric Association, 2013). Although ASD and ADHD differ (i.e., age of onset and typical introverted vs. extraverted profiles), the disorders do share many similarities. For example, ASD and ADHD show overlap in genetic risk factors (Ronald et al., 2008; Niklasson et al., 2009; Geschwind, 2011) and there is a high level of comorbidity between the two disorders (∼41–78% of ASD individuals experience ADHD symptoms; Clark et al., 1999; Simonoff et al., 2008; Murray, 2010; Rommelse et al., 2011; Antshel et al., 2013; van Steensel et al., 2013; Stevens et al., 2016). Both disorders also have a higher prevalence in boys than girls (Barkley, 2006; Bruchmüller et al., 2012; American Psychiatric Association, 2013; Loomes et al., 2017). Additionally, ASD and ADHD groups show impaired social cognition and suffer comparable deficits in executive functioning (Sinzig et al., 2008; Caillies et al., 2014; Mazza et al., 2014; Bora and Pantelis, 2016; Craig et al., 2016). Moreover, accumulating evidence suggests a considerable overlap in neural correlates between the two disorders. For example, ASD and ADHD show comparable diffusion tract results, levels of gyrification and white matter structure (Aoki et al., 2017; Kushki et al., 2019; Gharehgazlou et al., 2022).

Dysfunction of the DA and NE systems has often been suggested as the neurobiological mechanism underlying ADHD (e.g., Robbins and Arnsten, 2009; del Campo et al., 2011; Xing et al., 2016; Faraone, 2018). For example, genes coding important proteins for catecholaminergic neuromodulation have often been implicated in the disorder and current ADHD medication such as methylphenidate (MPH) targets (by blocking reuptake transporters) DA and NE (Stahl, 2013; Huss et al., 2017; Maia et al., 2017; Faraone, 2018; Myer et al., 2018; Faraone and Larsson, 2019). When compared to ASD, similar findings have been reported in regard to connectivity in ADHD: intraindividual variability of connectivity seems to be affected (Wang R. et al., 2015; Wang et al., 2018). More specifically, the DMN seems to show lower intraindividual variability in connectivity, while the rest of the brain shows higher intraindividual variability in connectivity (de Lacy and Calhoun, 2018). In a case study, Salgado et al. (2007) describe a patient with a brainstem lesion who showed ADHD symptoms (e.g., inattention). These symptoms could be ameliorated by MPH (Salgado et al., 2007). This illustrates the important role of the brainstem catecholamines in ADHD symptoms and how ADHD medication can help restore neuromodulation (see Johnston et al., 2014).

If ASD and ADHD are characterized by similar neuromodulatory disturbances, targeting the same neurotransmitter systems as current ADHD medication may prove helpful for the treatment of ASD. To illustrate more generally, such off-label application of stimulants has also proven valuable in other contexts such as subpopulations within major depressive disorder (Candy et al., 2008; Corp et al., 2014). In this review, catecholaminergic and cholinergic neuromodulation will be examined in relation to ASD and ADHD – with a focus on the SaN, cerebellum and genetics (to be further introduced later). These neuromodulatory systems will be described in detail and related to the symptomatology of ASD and ADHD. Subsequently, human trials with pharmacological agents targeting the catecholaminergic and cholinergic systems in ASD will be discussed. This will provide insight into which pharmacological agents could help the treatment of ASD and whether such treatments could be similar to the current pharmacological treatment of ADHD. Furthermore, this approach allows for personalizing treatments based on the underlying (dys)functioning of neuromodulatory systems, which is highly compatible with the now established Research Domain Criteria (RDoC) approach (see Pacheco et al., 2022).

## The locus coeruleus-norepinephrine neuromodulatory system

### Connectivity of the locus coeruleus

The LC is a bilateral nucleus in the brainstem containing ∼60,000 NE neurons that project throughout almost the entirety of the brain (Andeìn et al., 1966; Mouton et al., 1994; Aston-Jones and Cohen, 2005a; Szabadi, 2013; Aston-Jones and Waterhouse, 2016). Classically, the LC-NE system was thought to influence the level of arousal and was suggested to be important in the modulation of whole brain states (Jouvet, 1969; Berridge and Waterhouse, 2003). The widespread projections of the system are in line with such an idea. However, recent evidence shows that individual neurons in LC have idiosyncratic projections (Chandler et al., 2014; Kebschull et al., 2016). Furthermore, ensembles of LC-NE neurons can signal specific neural sites (reviewed in Totah et al., 2018; Chandler et al., 2019). This challenges the classical idea that the LC-NE system projects homogenously across neural sites and suggests that the system may be more flexible than previously thought.

Research in rodents has shown that ACC and prefrontal areas receive the most abundant LC-NE projections (Schwarz et al., 2015). Uniquely, insular cortex receives input from both LC-NE neurons and prefrontal NE neurons (Robertson et al., 2013). Using viral-genetic tracing methods, Schwarz et al. (2015) were able to elucidate afferent projections to LC. Afferent projections come from many sites including cortical areas and amygdala. Notably, Purkinje cells (PC) in the cerebellum provide a considerable fraction of the input into LC and do not project to any LC-NE output sites (Schwarz et al., 2015). In turn, LC seems to modulate cerebellar associative synaptic plasticity at climbing fiber-PC synapses (Carey and Regehr, 2009). This indicates reciprocal neural actions between LC and cerebellum.

### Norepinephrinergic neuromodulation in ASD and ADHD

In an extensive review, London (2018) described how the LC-NE system may be involved in the symptomatology of ASD (also see Mehler and Purpura, 2009). Although mostly based on indirect evidence, LC-NE system dysfunction has the potential to explain many symptoms and impairments reported in ASD and ADHD. Potential aberrant LC-NE system functioning in ASD can explain deficits in learning, attention, language, sensory processing and emotional functioning, but can also explain symptoms linked to the autonomic nervous system and sleeping problems (London, 2018). Individuals with ADHD show all of the impairments listed above, although these impairments differ substantially in severity (Cohen et al., 2000; Bruce et al., 2006; Geurts and Embrechts, 2008; Tonhajzerova et al., 2009; Konofal et al., 2010; Ghanizadeh, 2011; Musser et al., 2011; Craig et al., 2016; Gregory et al., 2017; Chevrier et al., 2019). The impairments mentioned above can be linked to the LC-NE system (London, 2018), which likely functions aberrantly in ADHD (Darcq and Kieffer, 2015; Xing et al., 2016; Faraone, 2018; Faraone and Larsson, 2019). Thus, potential impaired LC-NE system functioning can explain a wide range of symptoms and impairments that are reported in both ASD and ADHD. It is important to establish if, and in what way, the LC-NE system functions differently in ASD and ADHD when compared to the healthy population so that pharmacological targets may be identified.

### Molecular genetics

Genetically, some genes have been identified that may influence the development of the LC-NE system, which may be affected in ASD and ADHD. Dopamine β-hydroxylase (DBH) is an enzyme that catalyzes the conversion of DA into NE (Kaufman and Friedman, 1965; Stahl, 2013). Alterations of *DBH*, the gene coding DBH, can lead to a relative increase in DA levels and a relative decrease in NE levels, which is highly relevant for LC-NE functioning. *DBH* seems to play a role in ADHD (Wigg et al., 2002; Zhang et al., 2004; Tong et al., 2015; but see Inkster et al., 2004). For example, *DBH* gene variant rs129882 was found to be associated with ADHD in a large sample (Tong et al., 2015). In ASD, *DBH* has also been implicated and maternal levels of DBH seem to play a role (Robinson et al., 2001; Jones et al., 2004; Yrigollen et al., 2008; Jwaid et al., 2020). Furthermore, in an ASD sample, *DBH* was associated with both ASD and ADHD behaviors (Barrie et al., 2018). Another potentially relevant NE-related gene is *SLC6A2*, which codes for the NE reuptake transporter. Although some studies report associations between *SLC6A2* and ADHD (Bobb et al., 2005; Sengupta et al., 2012; Hawi et al., 2013), others report no such association (McEvoy et al., 2002; de Luca et al., 2004). Sengupta et al. (2012) suggest that sex, subtypes of ADHD phenotypes and specific haplotype blocks of the *SLC6A2* gene are important factors to consider (see Zhu et al., 2004). Other evidence shows that *SLC6A2* is important in identifying whether MPH can be an effective treatment for ADHD. Specifically, two polymorphisms, rs28386840 and rs5569, were found to be associated with decreased MPH efficacy in ADHD (Yang et al., 2004; Myer et al., 2018). The one study examining a relation between *SLC6A2* and ASD did not reveal a significant association (Park et al., 2014). Other work has also pointed toward epigenetic factors during early development in combination with LC-NE that may affect ASD symptom onset and severity (Mehler and Purpura, 2009). More specifically, it has been suggested that fever during this early period may restore LC-NE dysfunction, which in turn is thought to lead to decreased severity of ASD symptomatology.

### Purkinje cells and cerebellum

As stated, PCs in the cerebellum have substantial projections to the LC (Schwarz et al., 2015). Notably, the lower total number of PCs or decreased PC density, are some of the most replicable neurobiological findings in ASD (∼75% of ASD subjects show dysfunction/decreased number of PCs; Fatemi et al., 2002; Whitney et al., 2009; Passarelli et al., 2013; Hampson and Blatt, 2015). Some evidence indicates that PC numbers are also decreased in ADHD, but more research is necessary (Rout et al., 2012; Passarelli et al., 2013). As Rout et al. (2012) note, decreased PC numbers may only be observed in an ADHD subtype with cerebellar dysfunction as revealed from more global neuroimaging assessment (see Durston et al., 2011). Currently, the genetic underpinnings of PCs in ASD are still beginning to be understood (Hampson and Blatt, 2015). One study by Rout et al. (2012) reported increased serum levels of antibodies against glutamic acid decarboxylase 65 (GAD65) in ASD and ADHD groups when compared to control subjects (GAD65-antibodies were not present in any of the healthy subjects). GAD65 is important in γ-aminobutyric acid (GABA) synthesis. Serum from the ASD and ADHD groups was subsequently applied to mouse cerebellum, where the antibodies reacted with PCs. Application of these antibodies ultimately resulted in PC death (Mitoma et al., 2003; Rout et al., 2012). Increased antibodies against GAD65 may therefore contribute to PC dysfunction in ASD and ADHD, but research in larger samples is required. Aberrant afferent PC projections in ASD and perhaps ADHD to LC may lead to LC-NE dysfunction. However, the reverse is also conceivable. As stated, the LC modulates cerebellar associative synaptic plasticity, which influences PC firing (Carey and Regehr, 2009). Therefore, anomalous LC-NE functioning can lead to disturbed PC activity. The last option is that the reciprocal projections between LC and cerebellum in both directions are anomalous, which would also indicate LC-NE and PC/cerebellar dysfunction.

### Functional networks

Among its widespread projections, LC innervates two important nodes of the SaN: ACC and insular cortex (Seeley et al., 2007; Robertson et al., 2013; Schwarz et al., 2015). Neuromodulation may play an important role in SaN regulation since this network shows a high level of dynamic changes over time when compared to other networks (Chen et al., 2016). Dysfunction of SaN has been suggested to underlie ASD and has been implicated in ADHD (Uddin and Menon, 2009; Aboitiz et al., 2014; Sidlauskaite et al., 2016). Interestingly, when compared to ECN, DMN and SaN, similar functions have been proposed for the LC-NE system (Aston-Jones and Cohen, 2005a,b). According to Adaptive Gain Theory, the LC has two modes. A *phasic* mode in which phasic activity is relatively high and tonic activity is moderate and a *tonic* mode which shows the reverse pattern. When in *phasic* mode, metabolic resources are used to process task-relevant stimuli, and this improves current task performance. In contrast, when in *tonic* mode, metabolic resources are no longer used to focus on the task and is linked to distractibility. Importantly, in *tonic* mode, resources are used to identify salient stimuli in the environment (Aston-Jones and Cohen, 2005a,b; Aston-Jones and Waterhouse, 2016). Whereas ECN and LC’s *phasic* mode are important during task performance, DMN and SaN can be linked to LC’s *tonic* mode. Furthermore, Zerbi et al. (2019) reported altered levels of intrinsic connectivity within the DMN and SaN in rodents after LC activation. These data further implicate the role of the LC-NE system in network modulatory effects.

Altogether, accumulating evidence from genetic, network and cellular studies is emerging for aberrant functioning of the LC-NE system in ASD and ADHD. LC-NE dysfunction may explain numerous symptoms observed in both disorders and NE could therefore be a potential pharmacological target for ameliorating such symptoms. However, note that some of the presented evidence is somewhat indirect and more direct evidence is needed to precisely identify in what way the LC-NE system functions aberrantly in ASD and ADHD. One possibility is that mutual connections between LC and cerebellar PCs are instrumental in this respect, pointing to a possible efficacy of NE-directed treatment in a majority of ASD, and perhaps a minority of the ADHD population.

## The dopaminergic neuromodulatory system

### Connectivity of VTA and SNc

The DArgic neuromodulatory system has two important DArgic nuclei in the brainstem: the SNc and VTA (Mesulam, 2000; Beier et al., 2015). It is important to consider the differences between these two nuclei. Firstly, the SNc and VTA have different efferent and afferent projections (Watabe-Uchida et al., 2012). The SNc mainly innervates dorsal striatum (DS; Lerner et al., 2015; Morales and Margolis, 2017). Moreover, evidence shows that the medial SNc and lateral SNc have independent efferent pathways to medial and lateral DS, respectively (Lerner et al., 2015). In turn, lateral DS was found to project to SNc (Watabe-Uchida et al., 2012; Lerner et al., 2015). VTA was found to project to nucleus accumbens (NAc), PFC and amygdala (di Michele et al., 2005; Chandler et al., 2013; Beier et al., 2015, 2019; Morales and Margolis, 2017). Afferent projections to VTA come from many areas including NAc, amygdala and ventral pallidum (Watabe-Uchida et al., 2012; Beier et al., 2015, 2019).

### Dopaminergic neuromodulation in ASD and ADHD

Recently, the DA system has also been implicated in ASD. Pavãl (2017) posits that two DArgic pathways play an important role in the core deficits of ASD: the mesocorticolimbic (MCL) pathway and the nigrostriatal (NS) pathway. VTA projections to PFC and NAc make up the MCL-pathway. This pathway is important in reward processing and shows hypoactivation in ASD (Dichter et al., 2012b; reviewed in Pellissier et al., 2018). In ASD, the value of social rewards is thought to be greatly reduced, which results in a lack of social motivation (Dawson et al., 2005; Chevallier et al., 2012). Social motivation theory suggests that a lack of social motivation is the underlying cause of many social impairments in ASD (Chevallier et al., 2012; Dichter et al., 2012a). Thus, aberrant MCL-pathway functioning is thought to underlie the social impairments of ASD (Pavãl, 2017). However, note that others posit that dysfunction of the Social Brain Network (i.e., inferior frontal gyrus, amygdala and fusiform face area) can explain impaired social motivation (Misra, 2014), which would imply that social motivation can be impaired without MCL-pathway dysfunction. The NS-pathway consists of SNc projections to DS. This pathway plays an important role in motor aspects of goal-directed behavior (Lewis et al., 2007; Howe and Dombeck, 2016). For example, increased sensitivity of striatal and cerebellar DA D2 receptors underlies motor impairments in Parkinson’s disease (Rinne et al., 1990). Both optogenetic and pharmacological stimulation of the DA D1 receptor in the NS-pathway results in repetitive and stereotyped autistic-like behavior in mice (Lee et al., 2018). These data implicate the NS-pathway in the core behavioral deficit of ASD (Horev et al., 2011; Pavãl, 2017; Lee et al., 2018). However, accumulating evidence opposes the functional distinction between the MCL- and NS-pathways (Rossi et al., 2013; Ilango et al., 2014; Haber, 2016). For example, rodents will self-administer stimulation to the SNc, implying that this structure plays a role in reward, although not a part of the MCL-pathway (Ilango et al., 2014). Nonetheless, even if the pathways are not functionally distinct, evidence suggests that DArgic dysfunction may underlie the core symptoms of ASD.

It is generally accepted that dysfunction of the DA system plays a vital role in ADHD pathology (Levy and Swanson, 2001; Faraone et al., 2005; Tripp and Wickens, 2009; Faraone and Mick, 2010; Purper-Ouakil et al., 2011; Faraone, 2018). DA dysfunction in ADHD has been linked to deficits in executive functions and learning (Swanson, 2003; Silvetti et al., 2013) but also to inattention, hyperactivity and impulsivity (Swanson, 2003; Genro et al., 2010). The development of SN and VTA connectivity has been shown to be disturbed in ADHD (Tomasi and Volkow, 2012). Furthermore, dysfunction of the MCL-pathway has been associated with ADHD-behaviors (Stark et al., 2011) and the NS-pathway has been linked to hyperactivity in the disorder (Genro et al., 2010). These data indicate dysfunction of the MCL- and NS-pathways in ASD and ADHD.

### Molecular genetics

Irregularities in genes that are involved in the development of the DA system have been identified in ASD and ADHD. *SLC6A3* is a gene that codes for the DA reuptake transporter, which has a high density in striatum and NAc (Brooks, 2016; Salatino-Oliveira et al., 2018). This gene has often been implicated in ADHD (Gizer et al., 2009; Faraone et al., 2014; Faraone and Larsson, 2019). For example, a meta-analysis reported a relation between a polymorphism of the *SLC6A3* and increased DA transporter presence in striatal areas as determined by positron emission topography (PET; Faraone et al., 2014). This polymorphism has also been linked to ADHD in adults (Franke et al., 2010). With respect to ASD, although some contradictory results regarding *SLC6A3* involvement have been reported (e.g., Makkonen et al., 2008), most studies indicate that *SLC6A3* is associated with the disorder (Hamilton et al., 2013; Nguyen et al., 2014). Genes coding the different DA receptors in relation to ASD and ADHD have also been investigated. Strong evidence suggests *DRD4* and *DRD5* are associated with ADHD, while weaker evidence suggests an association between ADHD and receptor genes *DRD1* and *DRD2* (Gizer et al., 2009). Moreover, multiple studies report *DRD3* to have no association with ADHD (Faraone et al., 2005; Genro et al., 2010). In ASD, many DA receptor genes have been implicated. *DRD1, DRD2, DRD3, DRD4*, and *DRD5* have all been associated with the disorder (Hettinger, 2009; Gadow et al., 2010; Taurines et al., 2011; Staal et al., 2012; Nguyen et al., 2014). Furthermore, associations between polymorphisms in *DRD3*, *DRD4* and repetitive behaviors in ASD have been reported (Gadow et al., 2010; Staal et al., 2012). This directly links DA dysfunction to ASD symptomatology. Taurines et al. (2011) compared the mRNA expression of DRD4 and DRD5 between ASD, ADHD and control groups. The data showed lower DRD4-mRNA in ASD and ADHD groups. Moreover, the ASD group showed lower DRD5-mRNA when compared to the ADHD and control groups (Taurines et al., 2011). Additionally, recent work has underlined the potential importance of epigenetic factors in ADHD (Pineda-Cirera et al., 2019). Methylation of multiple genes (*ARTN*, *PIDD1*, and *C2orf82*) were found to be linked to expression of these genes, mainly in cerebellum, subcortical regions and frontal cortex, and this was predictive of ADHD. Importantly, these genes have been implicated in fetal and postnatal neurodevelopment including NAc, pointing toward involvement of epigenetic factors in the dysfunction of the DA system in ADHD (Pineda-Cirera et al., 2019). Altogether, genetic factors seem to contribute to dysfunction of the DA system in both ASD and ADHD. Although identified genetic loci in the DA system are shared between the two disorders, there are also important differences such as the role of *DRD3*.

### Functional networks

As discussed, the SaN has been implicated in ASD and ADHD symptomatology. The SN has been shown to be connected with the SaN in humans using diffusion tensor imaging (Zhang et al., 2017). Additionally, functional connectivity between SN, VTA and SaN nodes has been reported in an fMRI study (Seeley et al., 2007). In an extensive review, the importance of the DA system in SaN functioning has been described (Peters et al., 2016). The authors suggest that cortico-striatal-thalamic loop circuits can regulate SaN functioning (Alexander et al., 1986; Peters et al., 2016; McCutcheon et al., 2019). More specifically, VTA and rostral SN pars reticularis innervate thalamic and striatal areas, which subsequently modulate cortical nodes of the SaN (del Campo et al., 2011; Haber, 2016; Peters et al., 2016). In line with this, deep brain stimulation of NAc and SN was found to boost activity in cortical SaN nodes (Alexander et al., 1986; Peters et al., 2016). In turn, a PET study showed that repetitive transcranial magnetic stimulation of dorsal lateral PFC increased DArgic transmission in caudate nucleus and thalamus (Strafella et al., 2001). This shows that SaN nodes and subcortical areas can reciprocally activate one another (Alexander et al., 1986; Peters et al., 2016). Functionally, the DA system and SaN have been proposed to have similar roles. Contemporary studies suggest that DArgic modulation not only influences reward learning and goal-directed behavior, but also plays a role in directing attention toward salient stimuli in the environment (Horvitz, 2000; Koob and Volkow, 2010; Kroemer et al., 2014; Peters et al., 2016). Together, this illustrates the importance of the DA system in SaN functioning.

In sum, dysfunction of the DA system likely contributes to the symptomatology in ASD and ADHD. Whereas there is strong evidence for the relation between DA dysfunction and ADHD, such evidence is relatively still emerging for ASD. Nevertheless, genetic evidence strongly implicates DA dysfunction in both disorders as *DBH*, *SLC6A3* and DA receptor genes have been implicated in ASD and ADHD. Moreover, the influence of the DA system on the SaN can help explain symptomatology in ASD and ADHD. Importantly, the DA system can be targeted by available pharmacological treatments, which could improve the future treatment of ASD. Further research should focus on elucidating exactly how the DA system is altered in ASD and ADHD, so that specific targets within the system may be identified.

## The nucleus basalis-acetylcholine neuromodulatory system

### Connectivity of the nucleus basalis

The NBM is an important cholinergic nucleus positioned in the basal forebrain and has widespread projections across the brain (Mesulam et al., 1983; Mesulam and Geula, 1988; McCormick, 1989; Mesulam, 2000; Liu et al., 2015). There are two types of cholinergic receptors: nicotinic (nAChR) and muscarinic receptors (mAChR; Jensen et al., 2005; Stahl, 2013; Fuenzalida et al., 2016). Comparable to the LC-NE system, the cholinergic system has classically been thought to modulate arousal (Szerb, 1967; Phillis, 1968; Aston-Jones and Cohen, 2005a). More recently, this system has been implicated in memory and attentional functions (Mesulam, 2000; Arnold et al., 2002; Liu et al., 2015; Mueller et al., 2017), but also in cognitive flexibility and social communication (Ragozzino et al., 1998; Karvat and Kimchi, 2014; Wang L. et al., 2015; Liu et al., 2018). No viral-tracing genetic studies have been performed to precisely examine the topography of the NBM. Nonetheless, the connections of the NBM have been examined, primarily in animal studies. NBM has widespread cortical projections, which are relatively more ventral when compared to the DA and NE systems (Mesulam et al., 1983; Mesulam and Geula, 1988; Kenemans and Ramsey, 2013; Liu et al., 2015). Furthermore, NBM has been shown to be connected to frontal areas and visual cortex (Nagasaka et al., 2017; Huppeì-Gourgues et al., 2018). Additionally, connections between the NBM and ventral striatal areas have been described (Shu et al., 2019). Also, NBM and amygdala have reciprocal projections to one another (Woolf and Butcher, 1982; Mesulam et al., 1983; Mesulam, 2000; Aitta-aho et al., 2018).

### Cholinergic neuromodulation in ASD and ADHD

Cholinergic neuromodulation is also altered in ASD. Cholinergic neurons in the NBM have been shown to be altered in size, number and structure in ASD (Kemper and Bauman, 1998). Moreover, decreased levels of the ACh precursor choline have been reported in ASD (Sokol et al., 2002; Friedman et al., 2006). Furthermore, epibatidine - a marker for α4β2-nAChRs (Jensen et al., 2005) – shows altered binding in frontal, parietal, striatal and cerebellar regions in ASD groups (Perry et al., 2001; Lee et al., 2002; Martin-Ruiz et al., 2004; Ray et al., 2005; Deutsch et al., 2010; Mukaetova-Ladinska et al., 2010; Marotta et al., 2020). Notably, decreased cerebellar binding of α4-nAChRs may lead to PC loss (Lee et al., 2002). Additionally, multiple preclinical ASD mouse models appear to suffer cholinergic deficits (Artoni et al., 2019; Marotta et al., 2020). For example, the BTBR mouse model for ASD is an inbred mouse strain that is characterized by a deletion within the *Itpr3* gene (Meyza and Blanchard, 2017). BTBR mice show repetitive behaviors, impairments in social communication and aberrant nicotinic cholinergic neurotransmission (Wang L. et al., 2015). Nicotine administration in BTBR mice ameliorated these characteristic ASD-like behaviors (Wang L. et al., 2015). Another study in BTBR mice reported similar effects when donepezil – an acetylcholinesterase inhibitor - was administered (Karvat and Kimchi, 2014). Although mAChRs have not been investigated extensively in relation to ASD, one study did report a 30% decrease in M1 receptor binding in ASD (Perry et al., 2001; Lee et al., 2002; Mukaetova-Ladinska et al., 2010). More research is necessary to understand potential dysfunction of the muscarinic system in ASD.

The cholinergic system has also been implicated in ADHD. Compared to healthy subjects, there is a larger percentage of smokers among the ADHD population, accompanied with lower percentages of quitting smoking (Pomerleau et al., 1995; Moolchan et al., 2000). This increase of consuming nicotine-containing substances could be a form of self-medication among ADHD individuals (Levin and Rezvani, 2002; but this could also be due to elevated impulsivity in ADHD, see Sousa et al., 2011) – similar ideas have been proposed for schizophrenia (Kumari and Postma, 2005). As discussed briefly, individuals with ADHD show a wide range of impairments such as decreased inhibition, impulsivity and executive functioning. Impaired cholinergic neurotransmission has been suggested to play a role in these cognitive deficits in ADHD (Potter et al., 2006, 2014). For example, one placebo-controlled study showed that nicotine administration improved performance on the Stroop task and also decreased stop-signal reaction time (SSRT) in ADHD subjects (Potter and Newhouse, 2004). Logemann et al. (2014) could not replicate the nicotinic effect on SSRT in healthy subjects, but strong responders to nicotine did show an enhanced stop-P3 electroencephalogram component when nicotine was administered (Kenemans, 2015). The nicotinic system has also been suggested to be involved in impulsivity, further underlining the importance of the cholinergic system in ADHD (Ohmura et al., 2012). Similar to ASD, mAChRs have not yet been thoroughly investigated in relation to ADHD. Nonetheless, mAChRs do appear to have decreased binding capacity in ADHD (Johansson et al., 2013). Together, more research is needed to elucidate the role of mAChRs in ADHD.

### Molecular genetics

Based on an analysis of genome-wide association study data, cholinergic receptor genes have been identified as candidate genes in ASD (Lee et al., 2012). Specifically, *CHRNA7* [coding the α7-nAChR (Jensen et al., 2005)] has been associated with ASD, as has been *CHRFAM7A* (Bacchelli et al., 2015) – an exclusively human and highly polymorphic hybrid gene consisting of a duplicated portion of *CHRNA7* fused to exons A-E of *FAM7A* (Gault et al., 1998; Wall et al., 2009; Pinto et al., 2010; Casey et al., 2012; Huguet et al., 2013; Bacchelli et al., 2015; Gillentine and Schaaf, 2015; Gillentine et al., 2017). *CHRNA7* has also been implicated in ADHD (Gillentine and Schaaf, 2015; Sinkus et al., 2015; Valbonesi et al., 2015; Gillentine et al., 2017; Baccarin et al., 2020), but not all studies have not replicated this finding (Kent et al., 2001; Faraone et al., 2005; Ross, 2012). *CHRFAM7A* has also been associated with ADHD (Williams et al., 2012; Baccarin et al., 2020), but replication is required to confirm this association. Another gene, *CHRNA4*, codes for the α4-nAChR and has been identified as a candidate gene for ASD, but this gene seems to only play a role in specific cases (i.e., combinations with other genes or pathologies; Moessner et al., 2007; Wall et al., 2009; Oikonomakis et al., 2016). Conversely, evidence for the involvement of *CHRNA4* in ADHD is strong, as multiple studies have reported an association of ADHD with the gene (Todd et al., 2003; Lee et al., 2008; Wallis et al., 2009; Faraone and Mick, 2010; Mastronardi et al., 2016). Moreover, *CHRNA4* has been linked to attentional problems and to SaN functioning (Todd et al., 2003; Sadaghiani et al., 2017).

### Functional networks

The cholinergic system may play a role in SaN functioning. Striatum, ACC and insula have the highest density of α4β2-nAChRs in the brain as determined by PET (Gallezot et al., 2005; Picard et al., 2013). As stated, these areas are crucial SaN nodes (Seeley et al., 2007; Peters et al., 2016). Sadaghiani et al. (2017) reported that *CHRNA4* polymorphism rs1044396 increased activity in ACC, insula and anterior prefrontal areas. Of note, rs1044396 showed no activity-altering effects in DMN and ECN (Sadaghiani et al., 2017). As mentioned previously, the cholinergic system seems to be involved in inhibiting responses (Logemann et al., 2014; Kenemans, 2015). Nicotine administration improves performance on stopping tasks in ADHD (Potter and Newhouse, 2004; Potter et al., 2006). Interestingly, Peters et al. (2016) reported that stopping and SaN show a remarkable overlap in activated brain areas. Kenemans and Ramsey (2013) posit that the cholinergic system is involved in salience detection (Furey et al., 2008). For example, when physostigmine, an acetylcholinesterase inhibitor, is administered, visual cortex showed enhanced activity to the first (and novel) trial, but not to subsequent trials (Furey et al., 2000). Furthermore, amygdala has also been suggested to be involved in salience detection (Sander et al., 2003; Kenemans and Ramsey, 2013). Amygdala is innervated by both the NBM as well as non-NBM cholinergic nuclei in the brainstem (Aitta-aho et al., 2018). In turn, amygdala innervates multiple neuromodulatory nuclei such as NBM, LC, VTA and SNc (Cardinal et al., 2002; Watabe-Uchida et al., 2012; Beier et al., 2015, 2019; Schwarz et al., 2015). These connections of the amygdala allow this structure to modulate activity of multiple neuromodulatory nuclei which modulate SaN functioning.

In sum, cholinergic neurotransmission is affected by genetic and molecular factors in both ASD and ADHD. The direct genetic and molecular influences of nAChRs within the SaN as well as the indirect influences via the amygdala make the cholinergic system crucial for proper SaN functioning. Therefore, dysfunction of the cholinergic system may underlie SaN dysfunction in ASD and ADHD. Dysfunction in salience detection can explain a myriad of symptoms in these disorders and normalizing this system may be a viable target for pharmacological interventions.

## Pharmacological interventions in ASD

As reviewed, the catecholaminergic and cholinergic systems play a role in ASD and ADHD symptomatology. Pharmacological interventions can target these systems which may help alleviate symptoms (Stahl, 2013). Currently, the atypical antipsychotics risperidone and aripiprazole are the only FDA approved medications for ASD and these drugs are generally prescribed to treat irritability (Bonnot and Holzer, 2012). In practice however, other off-label medications such as stimulants are prescribed to treat ASD (Murray et al., 2014). It is important to consider how prescribed medications affect ASD symptomatology and if these medications are tolerable within the ASD population. Table 1 shows all current randomized and placebo-controlled pharmacological trials in human ASD subjects. Only drugs targeting the catecholaminergic and cholinergic–and sometimes serotonergic–systems are considered.

**TABLE 1:** Overview of human RCTs targeting DA, NE and/or ACh in ASD.

Drug	References	Participants	Age^a^	Outcome measures	Effect size (*d*_*av*_)^b^
Propranolol	Beversdorf et al., 2008	18 total: 9 ASD, 9 control	29.3 (9.9)	Verbal problem solving	0.41
	Beversdorf et al., 2011	28 total: 14 ASD, 14 control	18.9 (4.2)	Semantic verbal fluency; letter fluency	0.43; *n.s.*
	Beversdorf et al., 2013	–^c^	–^c^	Social functioning	sig.^c^
	Beversdorf et al., 2014	–^c^	–^c^	Social functioning	sig.^c^
	Bodner et al., 2012	27 total: 14 ASD, 13 control	18.9 (2.4)	Working memory	0.72
	Zamzow et al., 2014	28 total: 14 ASD, 14 control	18.3 (2.7)	Mouth fixation; eye fixation	0.54; *n.s.*
	Zamzow et al., 2016	20 ASD	21.4 (4.6)	Conversational reciprocity	0.40
	Zamzow et al., 2017	18 ASD	21.4 (4.6)	Verbal problem solving speed; accuracy	0.41; *n.s.*
Atomoxetine	Arnold et al., 2006	16 ASD	9.3 (2.9)	Hyperactivity; social withdrawal; hyperactive/impulsive symptoms	0.40; 0.34; 1.27
	Harfterkamp et al., 2012, 2014	97 ASD (all children)	10.0 (2.8)	ADHD total; inattention; hyperactive/impulsive symptoms; Hyperactivity; stereotypy	1.09; 0.66; 1.12; 1.01; 0.79
Clonidine (transdermal)	Fankhauser et al., 1992	9 ASD	12.9 (9.6)	Global Improvement rating; social relationships, affectual reactions, sensory responses	1.39; 1.54; 0.54; 1.33
Clonidine (oral)	Jaselskis et al., 1992	8 ASD	–^d^	Hyperactivity; irritability; stereotypy; inappropriate speech	0.32; 0.64; 0.24; 0.30
Guanfacine	Handen et al., 2008	11 ASD (all children)	7.3 (1.4)	Global rating of improvement; hyperactivity rated by parents; hyperactivity rated by teachers	1.33; 1.04; 1.34
	Scahill et al., 2015; Politte et al., 2018	62 ASD (all children)	8.5 (2.3)	Home situation; obsessive-compulsive symptoms; hyperactivity; stereotypy; inappropriate speech; inattention; ADHD rating	0.74; 0.57; 1.21; 0.29; 0.28; 0.97; 1.45
Methylphenidate	Handen et al., 2000	13 ASD with ADHD symptoms (all children)	7.4 (1.7)	Hyperactivity; inappropriate speech	1.34; 0.77
	Pearson et al., 2013	24 ASD with ADHD symptoms (all children)	8.8 (1.6)	Hyperactivity; irritability; inappropriate speech; CGI total rated by parents; rated by teachers	0.86; 0.62; 0.41; 1.00; 1.35
	Quintana et al., 1995	10 ASD with ADHD symptoms	8.5 (1.3)	Hyperactivity; irritability	0.63; 0.69
	Research Units on Pediatric Psychopharmacology Autism Network [RUPPAN], 2005	66 ASD with ADHD symptoms	7.5 (3.3)	Hyperactivity rated by parents; rated by teachers	0.40; 0.48
Haloperidol	Anderson et al., 1989	45 ASD (all children)	4.5 (1.2)	Discrimination learning; CPRS total; withdrawal; stereotypy; fidgetiness; hyperactivity; CGI total; temper outbursts	*n.s.*; rest sig.^d^
	Campbell et al., 1982; Anderson et al., 1984	40 ASD (all children)	4.6 (–^d^)	Discrimination learning; CPRS total; CGI total	0.46; rest sig. ^d^
	Remington et al., 2001	36 ASD	16.3 (6.4)	Hyperactivity; CARS total; irritability; social withdrawal; irritability; inappropriate speech	sig.^d^; rest *n.s*
Risperidone	Luby et al., 2006	24 ASD (all children)	4.0 (1.0)	CARS total	0.62
	McCracken et al., 2002; McDougle et al., 2005	101 ASD (all children)	8.8 (2.7)	Hyperactivity; irritability; stereotypy; inappropriate speech; Ritvo total; sensory motor behaviors; affectual reactions; sensory responses	1.05; 1.44; 0.66; 0.33; 0.94; 0.74; 0.88; 0.79
	McDougle et al., 1998	31 ASD (all adults)	28.1 (7.3)	CGI total; repetitive behavior; aggression (all after 12 weeks); sensory motor behaviors; affectual reactions; Ritvo total	0.41; 0.29; 0.39; 0.19; 0.45; 0.15
	Nagaraj et al., 2006	39 ASD (all children)	5.0 (1.7)	CARS total; CGAS total	sig.^d^; 1.06
	Shea et al., 2004	77 ASD (all children)	7.5 (2.3)	Hyperactivity; irritability; social withdrawal; inappropriate speech; conduct problems; insecure/anxious; overly sensitive	0.81; 0.61; 0.38; 0.28; 0.36; 0.14; 0.32
	Troost et al., 2005	24 ASD (all children)	9.1 (2.3)	Relapse of irritability	0.68
Aripiprazole	Findling et al., 2014	41 ASD (all children)	10.4 (2.8)	Relapse of irritability	*n.s.*
	Ichikawa et al., 2017	93 ASD (all children)	10.1 (3.2)	Hyperactivity; irritability; CGAS total; CGI total	0.71; 0.58; 0.35; 1.03
	Marcus et al., 2009	218 ASD (all children)	9.7 (3.1)	Hyperactivity; stereotypy; inappropriate speech; CGI total	0.86; 0.49; 0.31; 0.77
	Owen et al., 2009	75 ASD (all children)	9.2 (2.9)	Hyperactivity; irritability; CGI total; stereotypy; inappropriate speech	all sig.^d^
	Varni et al., 2012	316 ASD (all children)	9.5 (3.1)	Quality of life; emotional functioning; social functioning; cognitive functioning	0.68; 0.48; 0.61; 0.45
Olanzapine	Hollander et al., 2006	11 ASD (all children)	9.0 (2.5)	CGI total; irritability; aggression	all *n.s.*
Desipramine	Gordon et al., 1992	7 ASD (all children)	9.6 (4.4)	Hyperactivity; CPRS total; NIHM obsessive-compulsive rating score	0.72; *n.s.*; *n.s.*
	Gordon et al., 1993	12 ASD	–^c^	Hyperactivity; autistic symptoms; anger; compulsive/ritualized behaviors	sig.^c^; rest *n.s.*
Clomipramine	Gordon et al., 1992	7 ASD (all children)	9.6 (4.4)	CPRS total; NIHM obsessive-compulsive rating score	4.00; 1.13
	Gordon et al., 1993	12 ASD	–^c^	Hyperactivity; autistic symptoms; anger; compulsive/ritualized behaviors	all sig.^c^
	Sanchez et al., 1996	8 ASD (all children)	6.4 (1.4)	CGI total; CPRS total	all *n.s.*
	Remington et al., 2001	36 ASD	16.3 (6.4)	CARS total, hyperactivity; irritability; social withdrawal; irritability; inappropriate speech	all *n.s.*
Tianeptine	Niederhofer et al., 2003	12 ASD (all children)	7.3 (3.3)	Hyperactivity; irritability; inappropriate speech; inappropriate eye contact	0.24; 0.47; 0.60; 0.18
	Wichers et al., 2021	38 total: 19 ASD, 19 control	30 (11)	Sustained attention; inhibition/stopping	all *n.s.*
Donepezil	Chez et al., 2003	17 ASD (all children)	6.8 (1.9)	Expressive speech; receptive speech	0.19; 0.20
	Handen et al., 2011	31 ASD (all children)	11.6 (.^d^)	Executive functioning	*n.s.*
Rivastigmine	Chez et al., 2004	32 ASD	–^c^	Expressive speech; overall autistic behavior	all sig.^c^
Galantamine	Niederhofer et al., 2002	20 ASD (all boys)	7.4 (1.2)	Hyperactivity; irritability; inadequate eye contact; inappropriate speech	0.32; 0.55; 0.22; 0.55
DMXB-A	Olincy et al., 2016	2 ASD	50 and 24 years old	Inattention; social dysfunction	*No statistical testing performed due to low sample size*
Transdermal nicotine	Lewis et al., 2018	8 ASD	24.0 (2.6)	Hyperactivity; irritability; social withdrawal; stereotypy; inappropriate speech; aggression	all *n.s.*
Mecamylamine	Arnold et al., 2012	18 ASD (all children)	7.4 (2.6)	Hyperactivity; irritability; social withdrawal; stereotypy; inappropriate speech; repetitive behaviors; social responsiveness	all *n.s.*

^a^Data presented as Mean (SD).

^b^Effect size calculated using the following formulas:$\sqrt{C\,o\,h\,e\,n^{\prime }\,s\,d_{a\,v}=\frac{\left(M_{P\,o\,s\,t\,P\,l\,a\,c\,e\,b\,o}-M_{P\,r\,e\,P\,l\,a\,c\,e\,b\,o}\right)-\left(M_{P\,o\,s\,t\,D\,r\,u\,g}-M_{P\,r\,e\,D\,r\,u\,g}\right)}{\left(\left(S\,D_{P\,o\,s\,t\,P\,l\,a\,c\,e\,b\,o}+S\,D_{P\,r\,e\,P\,l\,a\,c\,e\,b\,o}+S\,D_{P\,o\,s\,r\,D\,r\,u\,g}+S\,D_{P\,r\,e\,D\,r\,u\,g}\right)/4\right)}}\,o\,r\,\sqrt{C\,o\,h\,e\,n^{\prime }\,s\,d_{a\,v}=\frac{\left(M_{P\,l\,a\,c\,e\,b\,o}-M_{D\,r\,u\,g}\right)}{\left(\left(S\,D_{P\,l\,a\,c\,e\,b\,o}+S\,D_{D\,r\,u\,g}\right)/2\right)}}$. See Lakens (2013).

^c^Article could not be accessed. Whenever possible, relevant information was retrieved from abstract.

^d^Relevant information to perform calculations was not provided.

n.s, non-signifiant; sig., significant; CARS, Childhood Autism Rating Scale; CGAS, Children’s Global Assessment Scale; CGI, Clinical Global Impression; CPRS, Children’s Psychiatric Rating Scale; NIHM, National Institute of Mental Health; Ritvo, Ritvo-Freeman real life rating scale; RUPPAN, Research Units on Pediatric Psychopharmacology Autism Network.

## Norepinephrinergic interventions in ASD

### Propranolol

Propranolol blocks NE β1 and β2 receptors in both the central and autonomic nervous systems (Mansur et al., 1998; Meyer and Quenzer, 2013). Multiple placebo-controlled studies have reported improvements in ASD, including normalization of facial scanning, enhanced (non-)verbal communication and improved social functioning (Beversdorf et al., 2013, 2014; Zamzow et al., 2014, 2016, 2017). Notably, one study showed that the effect of propranolol on verbal problem solving speed was mediated by heart rate variability and baseline anxiety measures (Zamzow et al., 2017). London et al. (2020) reported propranolol to lower aggressive and self-injurious behaviors, but the study was not placebo controlled. Additionally, while in some studies anxiety was unaffected, others reported a decrease in anxiety (Sagar-Ouriaghli et al., 2018). Furthermore, in one study propranolol administration boosted functional connectivity in the DMN in both control and ASD groups, while another study reports increased functional connectivity in language areas in the left hemisphere in ASD (Narayanan et al., 2010; Hegarty et al., 2017). Intraindividual variability of functional connectivity was not assessed in these studies. Although studies have investigated the effects of propranolol on specific symptoms of ASD (i.e., facial scanning and language), the core deficits (i.e., social impairments and repetitive and restricted behavior) have not often been assessed (but see Beversdorf et al., 2013; Beversdorf et al., 2014). Additionally, most propranolol trials have small sample sizes and do not directly assess outcome measures that are clinically relevant, such as irritability, ADHD symptomatology and stereotypical behavior. Since most of these studies were not full clinical trials but instead single dose pharmacological studies (Beversdorf et al., 2013, 2014; Zamzow et al., 2014, 2016, 2017), it is necessary to conduct double-blind placebo controlled trials to properly assess the possible effects of propranolol on the core deficits of ASD and to assess propranolol’s potential effects in clinical treatments.

### Atomoxetine

Atomoxetine is a selective norepinephrine reuptake inhibitor, effectively enhancing NErgic transmission (Ettinger, 2011). Multiple studies have investigated whether atomoxetine can ameliorate ADHD symptoms in ASD groups. All currently reviewed studies report beneficial effects of atomoxetine on hyperactivity, inattention and impulsive behavior (Arnold et al., 2006; Harfterkamp et al., 2012, 2013, 2014). One limitation of the current ASD atomoxetine trials is that most studies have investigated the effect of atomoxetine on ADHD symptoms in ASD, but ASD symptoms have often not been assessed (but see Harfterkamp et al., 2014). However, in a non-placebo controlled trial, Jou et al. (2005) report an improvement of general behavior (e.g., less deviant) after atomoxetine treatment when compared to before starting the treatment. Additionally, one trial reported that atomoxetine can decrease stereotypical behaviors in ASD (Harfterkamp et al., 2014). In conclusion, although atomoxetine seems to alleviate ADHD symptoms in ASD, it remains unclear whether atomoxetine can aid in the treatment of ASD symptomatology.

### Clonidine and guanfacine

Clonidine and guanfacine are α2 receptor agonists and operate mainly presynaptically (on autoreceptors; Meyer and Quenzer, 2013). Two double-blind placebo-controlled trials have assessed the potential therapeutic effects of clonidine – a net NE antagonist – in ASD. One trial showed that oral clonidine may help treat hyperactivity, irritability, stereotypy and inappropriate speech (Jaselskis et al., 1992). Another study, using transdermal clonidine, administration reported beneficial effects on global improvement, social relationships, affectual reactions and sensory responses (Fankhauser et al., 1992). These trials seem promising for clonidine as a treatment option, but they are limited by their small sample sizes, as well as the side effect profile of the drug (i.e., irritability and sedation).

Lastly, α2 receptor agonist guanfacine has recently been investigated in ASD, in part due to the recent interest in extended-release guanfacine and ADHD trials investigating the drug. Guanfacine seems to have a myriad of positive effects in ASD. The two existing trials report beneficial effects on hyperactivity, stereotypy, inappropriate speech, inattention, ADHD-behaviors, global ratings of improvement, compulsive behaviors as well as an improved home situation (Handen et al., 2008; Scahill et al., 2015; Politte et al., 2018). Adverse effects were limited (i.e., drowsiness) and tolerability was generally high (Scahill et al., 2015; Politte et al., 2018). It is important to note that both clonidine and guanfacine can show net NE agonistic or antagonistic effects based on the dose (Svensson et al., 1975). Future studies - and potentially clinicians – should take this dose-dependent effect into account since this may affect efficacy and tolerability of these substances.

## Dopaminergic interventions: Stimulants and (a)typical antipsychotics in ASD

### Stimulants

Individuals with ADHD are commonly prescribed stimulants such as MPH or amphetamines (Murray et al., 2014), which both block DA and NE reuptake, along with some other agonistic mechanisms for especially amphetamines. MPH also reportedly lowers ADHD symptoms such as hyperactivity, inattention and impulsivity in ASD (e.g., Quintana et al., 1995; Pearson et al., 2013). Nonetheless, individuals with ASD have a lower chance of responding to MPH and suffer from adverse effects more often than ADHD individuals (Handen et al., 2000; Research Units on Pediatric Psychopharmacology Autism Network [RUPPAN], 2005). McCracken et al. (2014) showed that efficacy and tolerability of MPH in ASD is mediated by genes: *DRD1, ADRA2, COMT, DRD3, DRD4, SLC6A3, SLC6A4, DRD2*, and *DRD3*. Similar genes mediate the efficacy of MPH in ADHD, as *SLC6A3, DRD4*, and *COMT* have been implicated (Myer et al., 2018). Although MPH increased social skills and the clinical global impression in ASD in some studies, a meta-analysis showed that MPH does not significantly affect the core deficits of ASD (Sturman et al., 2017). However, this could be due to the low number of studies assessing the core deficits or genetic mediation of efficacy and tolerability of MPH (McCracken et al., 2014).

### Antipsychotics

Haloperidol is a typical antipsychotic that mainly antagonizes DArgic neurotransmission by blocking the DA D2 receptor. Administration of haloperidol was found to decrease hyperactivity in two reports (Anderson et al., 1989; Remington et al., 2001). Furthermore, compared to placebo haloperidol beneficially impacted global impression and children’s psychiatry rating scale scores in two studies (Campbell et al., 1982; Anderson et al., 1984; Remington et al., 2001). Inconsistent results are found when examining discrimination learning. One trial reports a significant, albeit modest, increase in discrimination learning (Campbell et al., 1982; Anderson et al., 1984), while another trial reports no such effects (Anderson et al., 1984). Although, in one study haloperidol decreased ASD symptoms such as withdrawal and stereotypy (Anderson et al., 1989), these effects were not replicated in a later trial (Remington et al., 2001).

The atypical antipsychotics risperidone and aripiprazole seem to share therapeutic effects in ASD, along with partial overlap in their pharmacodynamic profiles (5-HT_2A_ antagonism for both, but partial D2 agonism for aripiprazole versus D2 (as well as α1) antagonism for risperidone). Both drugs decrease hyperactivity and irritability and both drugs seem to benefit the clinical global impression (Aman et al., 2010; Cohen et al., 2013; Table 1). Additionally, the drugs have overlapping side effects such as weight change, altered appetite, drowsiness and sedation. A trial comparing risperidone to aripiprazole in ASD revealed that the efficacy and tolerability are comparable (Ghanizadeh et al., 2014). Nonetheless, differences between the effects of risperidone and aripiprazole are also reported. While risperidone has been found to reduce aggression and autistic behaviors (McDougle et al., 1998; Luby et al., 2006; Nagaraj et al., 2006), aripiprazole improved quality of life (Varni et al., 2012). Although these atypical antipsychotics may reduce overall autistic behaviors in some studies, adverse effects are often problematic and these medications are not tolerable in many individuals (Orsolini et al., 2016). Genetic studies may help identify genes that mediate efficacy and tolerability of atypical antipsychotics in the treatment of ASD. Note that one trial investigating another atypical antipsychotic, olanzapine, showed no significant therapeutic effects (Hollander et al., 2006). Lastly, none of the reviewed studies have assessed the effects of MPH or typical antipsychotics on the DA MCL- and NS- pathways in ASD. Investigating such effects in ASD may provide insight into the potential therapeutic effects of these agents on the core deficits of the disorder (Pavãl, 2017).

## Cholinergic interventions and tricyclic antidepressants in ASD

### Cholinergic agents

Drugs targeting the cholinergic system in ASD have not been extensively researched. Many of these agents enhance cholinergic transmission by inactivating the acetylcholine-esterase enzyme (donepezil, rivastigmine) or by additional direct agonism of the nicotine receptor (galantamine; Stahl, 2008). The most researched cholinergic drug in relation to the disorder is donepezil. Donepezil reportedly increased both receptive and expressive speech, but did not influence executive functioning or overall autistic behaviors when compared to placebo (Chez et al., 2003; Handen et al., 2011). One placebo-controlled galantamine study has been conducted which reports improvements in hyperactivity, irritability, eye-contact and inappropriate speech (Niederhofer et al., 2002; also see Ghaleiha et al., 2014 where galantamine was added to the existing prescribed medication). Rivastigmine seems to improve expressive speech and overall autistic behaviors, but not enough research has been conducted to properly assess how these agents may benefit future treatment (Chez et al., 2004). A relatively new drug DMXB-A, an α7-nAChR agonist, improved neurocognition in schizophrenia (Olincy et al., 2006). In a sample of only two adults with ASD, the drug decreased inattention and decreased social dysfunction in one of these subjects (Olincy et al., 2016). Transdermal nicotine administration showed no significant effects in a placebo-controlled trial (Lewis et al., 2018; Table 1). The nicotinic antagonist mecamylamine also showed no significant effects in a placebo-controlled trial (Arnold et al., 2012; Table 1). As stated, cholinergic drugs have only been scarcely researched in relation to ASD. Double-blind placebo-controlled studies are necessary to understand how these agents can potentially aid future treatment of ASD.

### Tricyclic antidepressants

Tricyclic antidepressants (TCAs) enhance especially NE transmission, but also 5-HT transmission; in both cases this is effectuated through reuptake blocking and thought to underly the antidepressant effect. Of note, they also block muscarinic ACh receptors which accounts for most of their undesired side effects (Kenemans, 2017). TCAs have also been investigated in relation to ASD. Across two trials, desipramine seemed to decrease hyperactivity but compulsive and autistic behaviors remained unaffected (Gordon et al., 1992, 1993). In contrast, the same trials showed that clomipramine did decrease ASD symptoms, compulsive behaviors and also hyperactivity (Gordon et al., 1992, 1993). However, other clomipramine trials did not replicate such effects (Sanchez et al., 1996; Remington et al., 2001). Another TCA, tianeptine, seems to decrease hyperactivity, irritability, inappropriate speech and inappropriate eye contact (Niederhofer et al., 2003). A recent fMRI study showed that tianeptine did not behaviorally affect sustained attention or inhibition/stopping in ASD, but the drug did normalize ECN activation during tasks (Wichers et al., 2021). Overall, although some trials report beneficial effects of TCAs in ASD, most trials show little to no significant therapeutic effects of this class of medications.

## Discussion

As reviewed, dysfunction of catecholaminergic and cholinergic neuromodulation play a role in the symptomatology of ASD and ADHD (Faraone et al., 2005; Potter et al., 2006; Faraone and Mick, 2010; Pavãl, 2017; London, 2018). Importantly, the dysfunction of these neuromodulatory systems seem to share similarities - as well as differences - between both disorders at the level of genetics, functional networks and at the cellular level that may provide guidance for the development of biological/pharmacological treatment options.

Accumulating evidence suggests overlapping genetic contributions to potential dysfunction of the catecholaminergic and cholinergic systems in ASD and ADHD (Faraone et al., 2005; Nguyen et al., 2014; Bacchelli et al., 2015; Faraone and Larsson, 2019). However, note that many of the reported studies concerning the genetics of the catecholaminergic and cholinergic systems have limited effects. This in turn may be directly related to the effectiveness of either NE- or DA- or ACh-directed interventions in either population. Therefore, more specific, personalizing biomarkers may be useful in designing intervention strategies especially in relation to ASD. One further clue is that there are also differences in the genetic associations (*DRD3*, specific for ASD, and *SLC6A2*, specific for ADHD).

One further difference between ASD and ADHD in the dysfunction of neuromodulatory systems is the role of PCs. PC functioning is affected by especially by NE and ACh neuromodulatory systems, and has been implicated in ASD pathology, but less strongly in ADHD (Rout et al., 2012). Therefore, targeting PCs may be suitable for ASD, and a specific ADHD subgroup (see Durston et al., 2011). Given the role of the NE and ACh systems in PC function, it seems useful to further investigate NE- and ACh-targeting agents as effective treatments in ASD. Trials so far have yielded promising results, especially for the NE-reuptake inhibitor atomoxetine, for α2 agonist guanfacine and for ACh-esterase inhibitors. As said, a similar strategy could be beneficial for a subgroup within the ADHD population. Personalizing or biomarking this subgroup could perhaps be effectuated using specific assessments for cerebellar dysfunction. For ASD, especially drugs targeting the cholinergic system have been under-examined, while accumulating neurobiological evidence suggests that these drugs can potentially help treatment (Lippiello, 2006; Mukaetova-Ladinska et al., 2010). Furthermore the efficacy of propranolol in ASD on verbal problem solving seems to be mediated by heart rate variability and baseline anxiety measures, further underlining the need for personalizing treatments (Zamzow et al., 2017; Beversdorf, 2020). Additionally, future pharmacological trials should consider the core deficits of ASD, as these deficits are currently not often assessed. Future studies should also investigate the neurobiological effects of these agents in ASD patients, as preliminarily done for propranolol and tianeptine (Narayanan et al., 2010; Hegarty et al., 2017; Wichers et al., 2021). For example, the intraindividual variability of connectivity can be assessed to see how pharmacological agents may influence this potential underlying mechanism of ASD pathology (Falahpour et al., 2016).

As for stimulants, MPH is a generally effective medication in ADHD, but only few studies have assessed its effects on core ASD deficits. The efficacy and tolerability of MPH are mediated by genetic factors in both ASD and ADHD groups (McCracken et al., 2014; Myer et al., 2018). As mentioned, while some MPH trials show some improvements in ASD symptomatology, a meta-analysis showed that MPH does not significantly influence the core deficits of ASD (Sturman et al., 2017). However, as supported by the genetic mediation studies (McCracken et al., 2014; Myer et al., 2018), MPH may only be effective in specific ASD subgroups. One clue is the association between MPH effectiveness and varieties of the *DRD3* and *SLC6A2* genes, in both ASD and ADHD populations. Other biomarkers may also be predictive in ASD. For example, pupillometry measures have been used in ASD to identify dysfunction of the LC-NE and NBM-ACh systems (Lynch, 2018; Artoni et al., 2019; de Vries et al., 2021). In addition, future research could investigate the value of eye blink rate to assess DA functioning in ASD (Jongkees and Colzato, 2016). Alternatively subgroups could be created based on cognitive functioning (i.e., attention or working memory), which has proved useful in identifying and predicting ADHD severity as well as its prognosis (Musser and Raiker, 2019; Pacheco et al., 2022). Pupillometry, eye blink rate and cognitive functioning, in combination with gene studies, may help identify which pharmacological treatment may be most effective for a specific individual.

In line with this personalization of treatments, adopting the RDoC approach may prove useful (Pacheco et al., 2022). Classifying disorders not categorically, but instead on a continuous scale based on traits (or other biomarkers) may prove more useful in investigating and understanding these disorders. Such an approach has already been found to be fruitful in both cognitive as well as imaging studies (Aoki et al., 2017; Kushki et al., 2019; Musser and Raiker, 2019; Gharehgazlou et al., 2022; Pacheco et al., 2022). In these studies the continuous approach could help predict severity of symptoms as well as neural underpinning more effectively than when adopting the classical categorical approach (Aoki et al., 2017; Kushki et al., 2019; Musser and Raiker, 2019; Gharehgazlou et al., 2022; Pacheco et al., 2022). Future research should incorporate this approach by at least including a continuous measure of ASD/ADHD traits.

The current review has some limitations. First, not all potentially important neuromodulatory substances have been examined. Although not considered here, the histaminergic, serotoninergic and opioid systems have been implicated in ASD pathology (Eissa et al., 2018; Pellissier et al., 2018). Also, the current review has examined the neuromodulatory systems as more or less independent, while these systems interact. For example, the dorsal raphe nuclei project to VTA, and VTA projects to the LC (Beier et al., 2015; Morales and Margolis, 2017). Moreover, NE reuptake transporters also clear DA from synapses in frontal areas (Madras et al., 2005). This illustrates how these systems are connected to one another and that the interactions between these systems should be investigated further (Marder, 2012).

Altogether, DA, NE, and ACh are important systems in ASD and ADHD symptomatology. Although the dysfunction of these systems show overlap between the disorders, there are also differences such as PC/cerebellar functioning and the involvement of specific genes (*DRD3, SLC6A2*). Current medications prescribed for ADHD are effective in ADHD groups, but there is currently not enough evidence to suggest that these medications can consistently and effectively alleviate core symptoms in ASD groups. NErgic agents, MPH, and cholinergic agents should be investigated further using double-blind placebo-controlled trials to confirm the potential therapeutic value of these agents and to assess their neurobiological effects in ASD. Lastly, biomarkers such as pupillometry, eye blink rate, cognitive functioning and genetics should be investigated in relation to ASD to identify which medications may be most effective for a specific individual.

## Author contributions

DK wrote the original draft of the manuscript. PD and JK provided the critical comments. DK and JK revised the manuscript. All authors contributed to the article and approved the submitted version.

## Footnotes

1 Although some contemporary work uses the term neuromodulation in relation to transcranial magnetic stimulation, transcranial direct-current stimulation or implants to modulate neural activity (e.g., Sokhadze et al., 2016; Nobusako et al., 2017; Krames et al., 2018), here we use this term in relation to neurotransmitters in the brain.
